# Acuity circles allocation policy impact on waitlist mortality and donation after circulatory death liver transplantation: A nationwide retrospective analysis

**DOI:** 10.1002/hsr2.1066

**Published:** 2023-02-01

**Authors:** Emmanouil Giorgakis, Tommy Ivanics, David Wallace, Allison Wells, Julius Balogh, Hailey Hardgrave, Derek Krinock, Garrett Klutts, Lyle Burdine, Andrew Singer, Amit Mathur

**Affiliations:** ^1^ Division of Transplant Surgery University of Arkansas for Medical Sciences Little Rock Arkansas USA; ^2^ Multi‐Organ Transplant Department Universit Health Network Toronto Canada; ^3^ Institute of Liver Studies King's College Hospital London UK; ^4^ Department of Anesthesiology University of Arkansas for Medical Sciences Little Rock Arkansas USA; ^5^ Department of Surgery University of Arkansas for Medical Sciences Little Rock Arkansas USA; ^6^ Division of Transplant Surgery Mayo Clinic Phoenix Arizona USA

**Keywords:** acuity circles, donation after circulatory death, liver allocation policy, liver transplantation

## 1 INTRODUCTION

The acuity circles (AC) liver distribution policy, implemented in February 2020 in the United States, was intended to address concerns over geographic variability in the median model for end‐stage liver disease at transplant (MMaT) across the country.^1^, ^2^, ^3^, ^4^ The AC model induced broader sharing of liver allografts, using concentric circles with the donor hospital at its center, rather than relying on arbitrary boundaries designated by the Organ Procurement and Transplantation Network region.^1^ In addition, the new policy was designed so that donation after circulatory death (DCD) offers were to be prioritized locally, that is, within 150 nautical miles radius from the donor hospital, thus expediting DCD allograft utilization and mitigating the effect of prolonged cold ischemia time on DCD outcomes.^1^, ^5^, ^6^, ^7^, ^8^, ^9^, ^10^, ^11^

Concerns were raised about the impact of the AC model on organ access due to increased logistical and financial burdens, which may further marginalize socioeconomically disadvantaged groups.^3^, ^5^, ^10^, ^12^, ^13^ Following its advent, the AC model predictably led to a net export of donation after brainstem death (DBD) allografts from low MMaT areas to their higher MMaT neighbors. We theorized that low MMaT centers experienced Darwinian pressure^14^ to adjust organ acceptance practices to manage their enlisted patients. High MMaT centers, in contrast, did not have these same pressures, as an increased influx of good quality organs may disincentivize utilization of DCD or other marginal liver allografts.

The aim of this study was primarily to explore the AC allocation policy's effect on DCD liver transplant (LT) rates and, secondarily, to assess the effect of the AC policy on waitlist mortality (WLM) in the United States.

## 2 METHODS

Aggregate data for all US DCD LT performed from January 2016 to July 2021 were retrieved following a request from the United Network for Organ Sharing. The DCD transplant rate, *defined as DCD LT performed per waitlisted patients per year*, was calculated for each of the 50 States (multiple transplant programs are present in most US States). The annual number of waitlisted patients and waitlist removals due to death were used to calculate State‐specific WLM. The cohort was dichotomized into pre‐AC (2016–2019) and AC (2020–2021) eras. The mean DCD LT and WLM were calculated for the pre‐AC and AC eras for each State. The State‐specific change (Δ) in DCD rate (ΔDCD) and WLM (ΔWLM) for the two eras were also calculated. To determine if there was a significant difference between the pre‐AC and AC DCD transplant rates, a two‐tailed *t*‐test was performed. A Pearson's correlation test was performed to assess for a directional association between continuous variables ΔWLM and ΔDCD (R Core Team, 2013) for the overall time period and then, specifically, for the AC period, using R software version 4.1.0.^15^, ^16^, ^17^ An *α* level of 0.05 was considered significant. Heat maps were built using the Excel Option for United States Mapping. The study was exempt from the institutional review board due to its deidentified registry nature and publicly retrievable data since in the United States such studies are not considered human subjects research.^18^ All statistical analysis was performed using IBM Statistical Package for the Social Sciences Statistic v25 (IBM Corp.).

## 3 RESULTS

**Figure 1:**
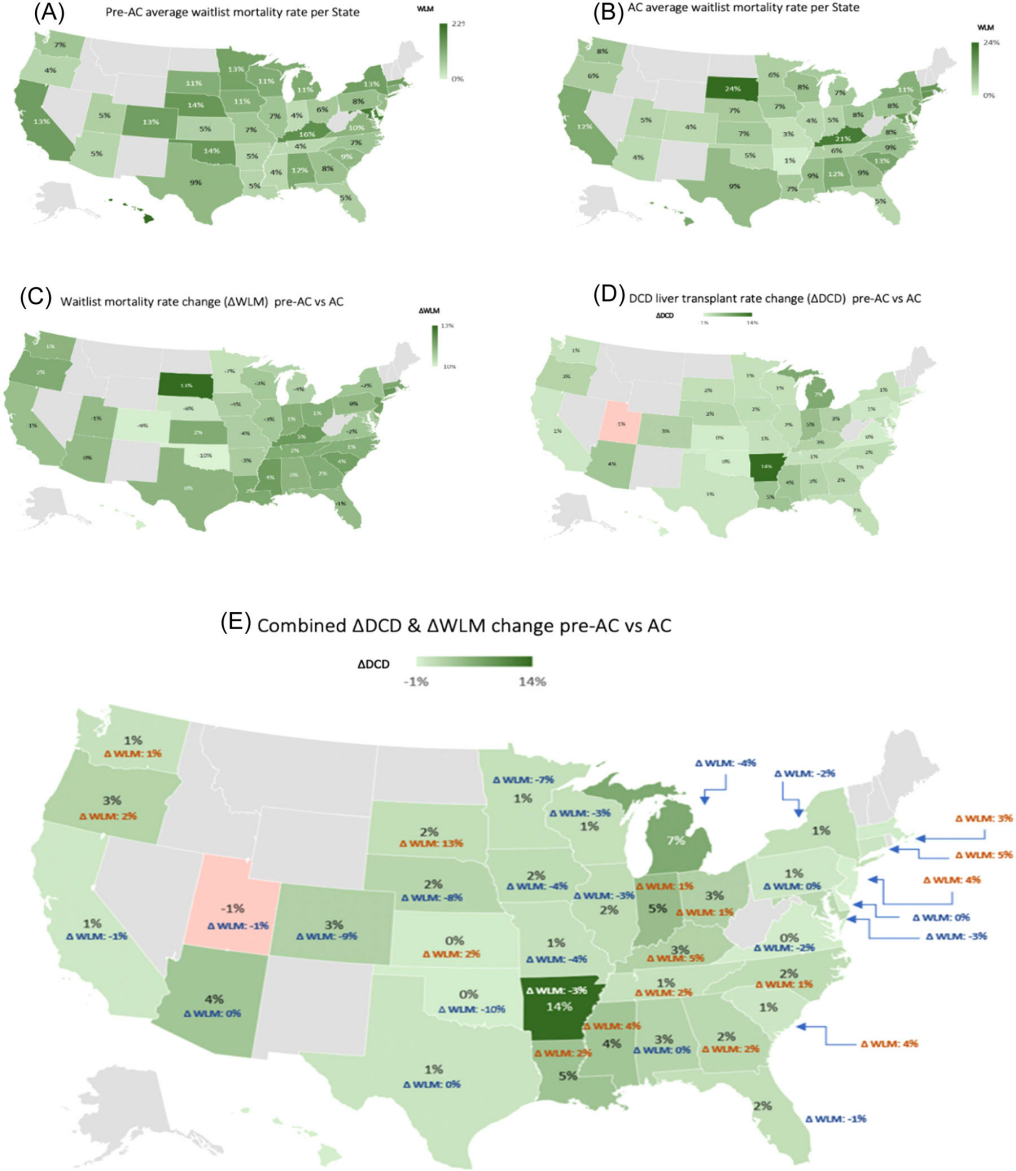
United States waitlist mortality and donation after circulatory death (DCD) liver transplant rate State heatmaps. (A) Pre‐acuity circles (AC) average annual waitlist mortality per State. (B) AC average annual waitlist mortality per State. (C) Waitlist mortality change (ΔWLM), pre‐AC versus AC eras. (D) DCD liver transplant rate change (ΔDCD), pre‐AC versus AC eras. (E) combined ΔWLM&ΔWLM, pre‐AC versus AC eras. DCD rate was defined as annual DCD liver transplants performed per waitlisted patients within the same State. ΔDCD, delta DCD rate; ΔWLM, delta waitlist mortality.

**Figure 2:**
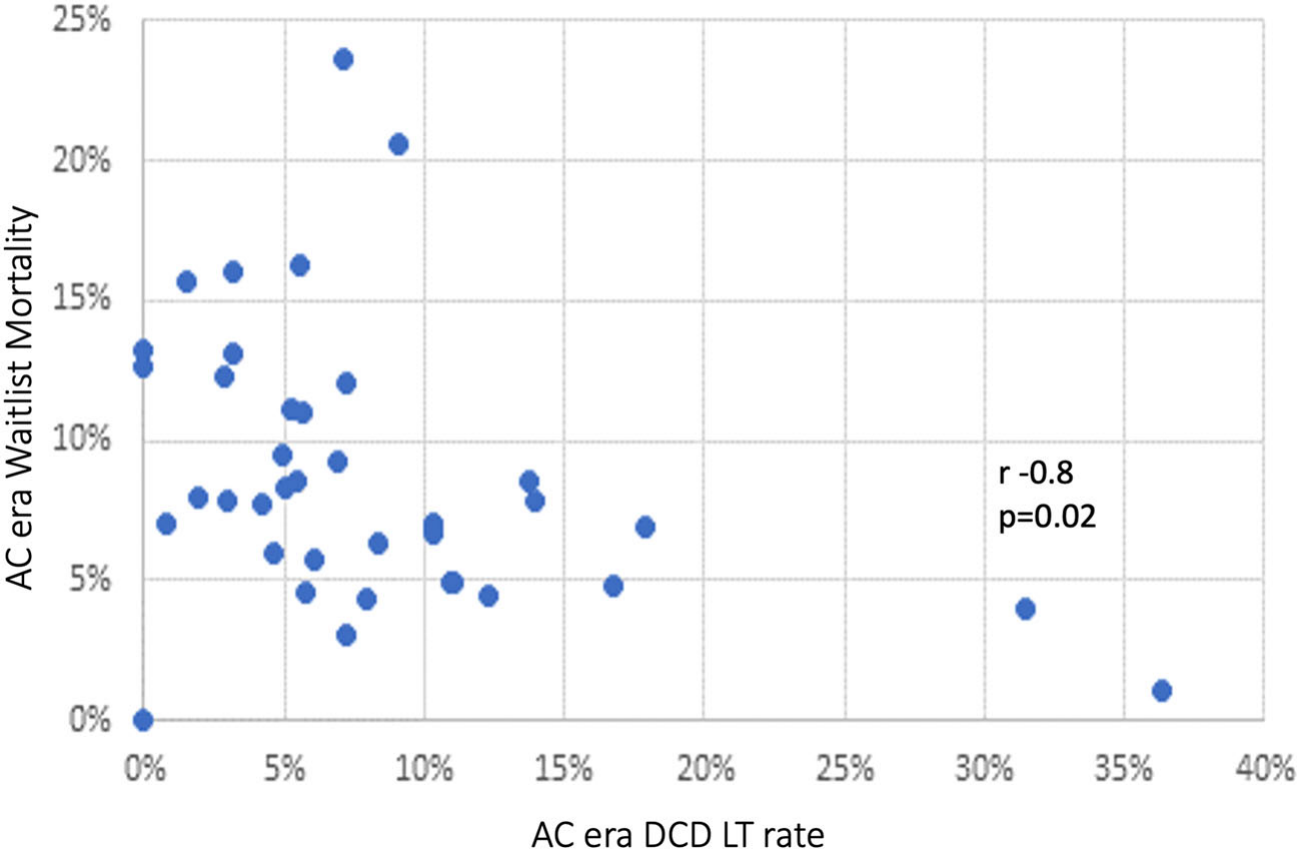
Pearson's correlation scatterplot of liver waitlist mortality and DCD LT rate during the acuity circle (AC) era. For the purposes of the study, DCD LT rate was defined as DCD LTs performed per waitlisted patients within the same State. There was a strong negative correlation (*r* = −0.8, *p* = 0.02) between the DCD LT rate and waitlist mortality. For the short AC period studied, waitlist mortality was less than 10% in States with DCD rates >10%. Waitlist mortality dropped to ≤5% in States with 30% DCD rates. Waitlist mortality dropped to ≤2% for DCD rate >35%. Data indicate that DCD LT can offer waitlist survival benefit to waitlisted patients that do not attract brain‐dead donor offers but remain suitable DCD LT candidates. DCD, donation after circulatory death; LT, liver transplant.

Since the implementation of the new allocation policy, there was wide WLM variation between States (−10% to +13% per year), while the nationwide average WLM remained largely unchanged (9%/year) (Figure 1A–C; Supporting Information: Data 1). Total DCD transplant rates were similar at 6% pre‐AC (6%/year) and AC (8%/year) (*p* = 0.15) but demonstrated wide variation among States (−1% to +14%) (Supporting Information: Data 2). The sharpest annual DCD rate gain was noted in Arkansas (14%), Louisiana (5%), Arizona (4%), and Mississippi (4%). DCD rate gains were minimal (0%–2%) in the densely populated northern coastal States. The highest absolute DCD rate was met in Arkansas (36%) and Arizona (31%), with ΔWLM −3% and 0%, respectively (Figure 1D,E; Supporting Information: Data 1 and 2). DCD LT rates and WLM were negatively correlated in the AC era (*r* = −0.38, *p* = 0.02). States that achieved the highest DCD rates managed to sustain or reduce their WLM (Figures 1E and 2).

## 4 DISCUSSION

At least for the limited period studied, nationwide WLM remained unchanged since the AC advent, a finding reported by others.^19^ However, WLM varied tremendously across States.

There is wide variation in DCD allograft utilization across the United States with a few centers driving national volume across the country.^20^, ^21^ Since AC, there has been an upward trend in US DCD liver utilization.^2^ This trend was not uniform across States and was spearheaded by a handful of centers, mostly serving low MMaT areas. Most of these States have vulnerable populations with higher rates of poverty and socioeconomic challenges. Based on the present data, transplant centers within these States have responded to an “evolutionary pressure,” triggered by a relative scarcity of local DBD donor livers that are now being distributed elsewhere. Some of these centers have been able to respond to the increased logistic challenges and costs associated with pursuing DCD donors.^10^

This brief report has various limitations. First, the effect of the AC policy is still taking shape, with institutions still adapting to the change. Second, this analysis is subject to multiple concurrent temporal confounders, including the opioid crisis which has been associated with higher donation volume, but also the COVID‐19 pandemic which has affected transplantation globally in an erratic manner. These factors confound attempts to attribute causation to the policy itself. Third, the comparisons were across States and not individual centers; as such, the analysis did not reflect individual transplant center activity (except for States with a single abdominal transplant center, e.g., Arkansas); therefore, it may not do justice to high‐volume DCD transplant champions that may outperform their respective state DCD average. Fourth, machine preservation technologies hold promise in optimization and more extensive use of extended criteria organs, such as DCD livers^22^; however, its impact on DCD utilization in the post‐AC era has not been explored. Lastly, the analysis did not consider DCD donor cases that did not result in a LT due to donor nonprogression or organ discard.

In conclusion, we found that States with a sharp rise in their DCD rates following the AC allocation policy advent managed to maintain or even increase their LT volumes while safeguarding their WLM, despite the drop in locally available DBD organs. This is likely related to center adaptability within these States to commit resources to DCD donation and utilization. Given that DCD LT can address WLM with reasonable risk‐adjusted outcomes, current trends indicate gross underutilization of DCD livers in the United States.

## AUTHOR CONTRIBUTIONS

**Emmanouil Giorgakis**: Conceptualization; funding acquisition; investigation; methodology; project administration; resources; supervision; validation; visualization; writing – original draft; writing – review and editing. **Tommy Ivanics**: Methodology; writing – review and editing. **David Wallace**: Writing – review and editing. **Allison Wells**: Data curation; formal analysis; methodology; software. **Julius Balogh**: Writing – review and editing. **Hailey Hardgrave**: Writing – review and editing. **Derek Krinock**: Writing – review and editing. **Garrett Klutts**: Writing – review and editing. **Lyle Burdine**: Funding acquisition; resources; writing – review and editing. **Andrew Singer**: Writing – review and editing. **Amit Mathur**: Methodology; writing – review and editing. All authors have read and approved the final version of the manuscript.

## CONFLICT OF INTEREST STATEMENT

The authors declare no conflict of interest.

## TRANSPARENCY STATEMENT

The lead author Emmanouil Giorgakis affirms that this manuscript is an honest, accurate, and transparent account of the study being reported; that no important aspects of the study have been omitted; and that any discrepancies from the study as planned (and, if relevant, registered) have been explained.

## Supporting information

Supporting information.

Supporting information.

## Data Availability

The authors confirm that the data supporting the findings of this study are available in publicly retrievable databases and Supporting Information Materials. Emmanouil Giorgakis had full access to all of the data in this study and takes complete responsibility for the integrity of the data and the accuracy of the data analysis.
